# Association Between the Results of the Timed Up-and-Go Test Adjusted for Disease Severity and Sarcopenia in Patients with Chronic Obstructive Pulmonary Disease: a Pilot Study

**DOI:** 10.6061/clinics/2019/e930

**Published:** 2019-10-30

**Authors:** Demetria Kovelis, Anna Raquel Silveira Gomes, Camila Mazzarin, Andrieli de Miranda, Silvia Valderramas

**Affiliations:** I Departamento de Fisioterapia, Universidade Dom Bosco (UniDBSCO), Curitiba, PR, BR; II Departamento de Prevencao e Reabilitacao em Fisioterapia, Universidade Federal do Parana (UFPR), Curitiba, PR, BR; III Programa de Pos-graduacao em Educacao Fisica, Universidade Federal do Parana (UFPR), Curitiba, PR, BR; IV Programa de Mestrado em Medicina Interna, Universidade Federal do Parana (UFPR), Curitiba, PR, BR; V Programa de Pos-graduacao em Medicina Interna, Universidade Federal do Parana (UFPR), Curitiba, PR, BR

**Keywords:** Lung Diseases, Hand Strength, Walking Speed, Sarcopenia, Physical Functional Performance

## Abstract

**OBJECTIVES:**

Loss of muscle mass and/or physical performance, a condition commonly known as sarcopenia, is prevalent in chronic obstructive pulmonary disease (COPD) and is associated with adverse outcomes. The aim of this study was to investigate the association between functional performance and sarcopenia in COPD patients classified by disease severity according to the Global Initiative for Chronic Obstructive Lung Disease (GOLD) criteria.

**METHODS:**

The study was a cross-sectional observational and the sample size consisted of 35 COPD patients (69.24±1.54 years, 20 men). Physical performance was assessed with the timed up-and-go (TUG) test. Sarcopenia was assessed according to the European Working Group on Sarcopenia in Older People criteria.

**RESULTS:**

The frequency of sarcopenia was 20% and was more prevalent among individuals classified with greater disease severity, GOLD III, n=4 patients (23%) and GOLD IV, n=3 patients (27%), *p*=0.83. The mean time to complete the TUG test was 11.66±4.78 s. Binary logistic regression revealed an association between the TUG test and sarcopenia adjusted by disease severity (OR=1.55, 95% CI: 1.03-8.23, *p*=0.04).

**CONCLUSION:**

Our findings showed that worse performance in the TUG test leads to a substantial increase in the chance of COPD patients presenting sarcopenia.

## INTRODUCTION

Chronic obstructive pulmonary disease (COPD) affects the lungs primarily by limiting airflow, and in more advanced stages of the disease, patients present dyspnea and reduced exercise tolerance (1). COPD is considered a systemic disease and is characterized by various extrapulmonary effects, including systemic inflammation (2), comorbidities (3) and skeletal muscle dysfunction, especially in the lower limbs (4). These effects contribute to a reduction in functional capacity (5) and impair the patient’s quality of life and ability to perform activities of daily living (6). In addition, the lack of infrastructure, social influences (7) and even the number of hours for which daily oxygen therapy (with equipment that limits the patient’s movements) is used can reduce the level of physical activity during daily living, leading in turn to a significant reduction in functional and exercise capacity in these patients (8).

Sarcopenia is another systemic manifestation in COPD (9,10). According to the European Working Group on Sarcopenia in Older People, sarcopenia is probable when low muscle strength is detected, and it is confirmed by the presence of low muscle quantity or quality. Furthermore, when low muscle strength, low muscle quantity/quality and low physical performance are all detected, sarcopenia is considered severe (11) and was recently recognized as an independent condition by the International Classification of Disease (12). In addition, as sarcopenia is strongly associated with multiple negative outcomes in the elderly, an investigation of this condition in COPD patients is fundamental to alert physicians for early diagnosis and treatment of loss of lean muscle mass, preventing worse outcomes and poorer quality of life (9,10).

The frequency of sarcopenia in COPD patients varies from 15% to 40% according to age and the method used to assess muscle mass, such as dual-energy X-ray absorptiometry (DXA) (9), bioimpedance analysis (10) and calf circumference (10-17). To date, few studies (10,18) have examined sarcopenia in COPD with an emphasis on the loss of muscle mass and physical performance. The results of these studies also support an association between sarcopenia and systemic inflammation, dyspnea (15), poor quality of life (10), poor prognosis (9) and frailty (19).

However, to our knowledge, the association between sarcopenia and the results of simple physical performance tests, such as the timed up-and-go (TUG) test, have not yet been investigated. Timed up and go test is reliable, valid and responsive (20) and has been used with COPD patients, as it can be performed in most healthcare settings. In our study, we hypothesized that COPD patients with sarcopenia might have worse performance in the TUG test when adjusted by disease severity. The aim of this study was to investigate the association between physical performance and sarcopenia in COPD patients classified by disease severity according to the GOLD criteria.

## MATERIALS AND METHODS

The study was a cross-sectional observational and was carried out between May 2016 and May 2017 following the recommendations of the Strengthening the Reporting of Observational Studies in Epidemiology (STROBE) initiative (21). The sample consisted of patients referred to a pulmonary rehabilitation program at a university center in the city of Curitiba in southern Brazil. After study approval by the ethics committee of the local municipal department of health (ref. no. 481.008/2013), potential participants were contacted by telephone to schedule an assessment. Individuals who were considered eligible signed a voluntary informed consent form that explained the aims, procedures, possible risks and benefits of the study. Patients were assessed at a physical therapy clinic or in their homes if they were unable to attend the clinic. All assessments were supervised by two previously trained physical therapists.

The inclusion criteria were as follows: being over 50 years of age; having a diagnosis of COPD classified as stage II to IV according to the GOLD criteria; having given up smoking at least 6 months previously; being clinically stable (i.e., the disease had been stable for at least 1 month); and not having been in any physical rehabilitation or training program in the previous year. Exclusion criteria were the presence of other pulmonary diseases (e.g., asthma and pulmonary fibrosis) or other incapacitating, severe or difficult-to-control nonpulmonary diseases that could interfere with the tests.

Pulmonary function was assessed with a spirometer (Microlab Spiro V 1.30, Micro Medical Ltd., Rochester, Kent, England) using the American Thoracic Society guidelines (ATS) (22) considering reference values established by Pereira et al. (23) Severity of airway obstruction was classified according to the GOLD criteria (GOLD II: 50% ≤VEF_1_ <80%; GOLD III: 30% ≤VEF_1_ <50%; GOLD IV <30%) (1).

### Primary Outcome

Physical performance was assessed with the TUG test (24,25). In the TUG test, the patient got up from a chair (seat height 45 cm) without using his arms, walked at a comfortable, safe pace for three meters, returned to the chair and sit down. The test was timed (in seconds) from the beginning of the test to the moment the participant leaned against the back of the chair again. TUG was first demonstrated by the researchers after the patient performed the test once to become familiar and then it was carried out again for timing (24). A cutoff point of 11.2s was used to indicate reduced functional performance (26).

### Secondary Outcome

The presence of sarcopenia was investigated by evaluating the handgrip strength (HGS), calf circumference and gait speed test (11).

HGS was assessed using a Saehan hydraulic hand dynamometer (Saehan Corporation - SH5001). The participant was seated with his feet on the ground, hips and knees flexed at 90° without any support for the arms, shoulders in adduction with neutral rotation and one elbow flexed at 90° with the ipsilateral forearm and wrist in neutral position. Then, the patient was asked to perform three maximal contractions with a 1-minute rest between each. The mean of the grip strength measurements in kilograms for the three attempts was used for analysis. Low HGS was defined as HGS <30 kg for men and <20 kg for women (27).

Skeletal muscle mass was estimated based on calf circumference and was measured in the most prominent region of the dominant calf with the patient sitting and the hip and knee flexed at 90°. A cutoff point of 30 cm was used to indicate reduced skeletal muscle mass (28).

Physical performance was assessed using the GS test, which was performed over 10 m, covered surface with positions marked on the floor at 0, 2, 4, 6, 8 and 10 m. Participants were positioned at the 0 m point and instructed to walk in a straight line for 10 m after the command “Go” was given. The distance to cover the route was divided by the time to give the mean (m/s). The test was performed three times, and the best value was used for analysis. The first and last 2 m were excluded to allow time for the patient to accelerate and decelerate (29,30). Participants were asked to walk at their normal pace, and no incentive was given to avoid influencing the result (29,30). A cutoff point of 0.8 m/s was used (31).

### Statistical Analysis

Sampling was nonprobabilistic, and all the patients who met the inclusion criteria in the study period were included. However, a post hoc calculation using an effect size of 0.5 based on Cohen’s d and the sample size of 35 patients showed a power of 91% to detect an association between TUG and sarcopenia in patients classified according to disease severity.

The Shapiro-Wilk test was used to test normality. The groups were compared by one-way analysis of variance (ANOVA) followed by Tukey’s post hoc test.

After adjustment of the results for disease severity according to the GOLD criteria, binary logistic regression was used to detect an association between the results of the TUG test and the presence of sarcopenia according to disease severity. A significance level of *p*<0.05 was used. The data were analyzed with Statistical Package for Social Sciences (SPSS) version 21.0, and study power was calculated with G* Power 3.1^®^.

## RESULTS

**Figure 1:**
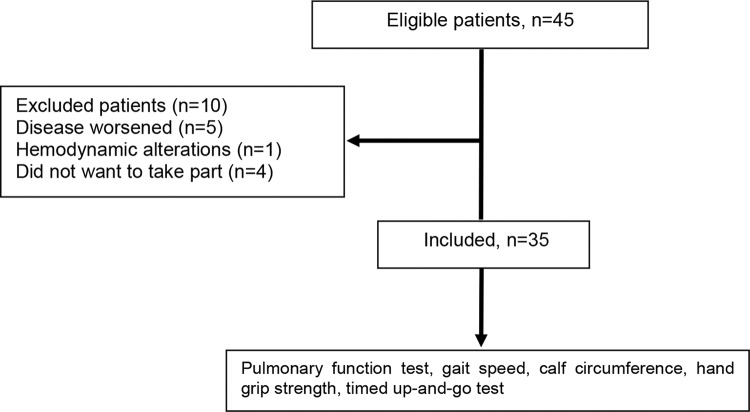
Data collection flowchart.

Forty-five individuals with COPD were recruited, but only 35 of these were included in the study, as shown in Figure 1. The demographic, anthropometric, clinical and functional characteristics of the patients are shown in Table 1. The patients were seniors, and the majority were classified as GOLD III (49%) or GOLD IV (31%).

**Figure 2:**
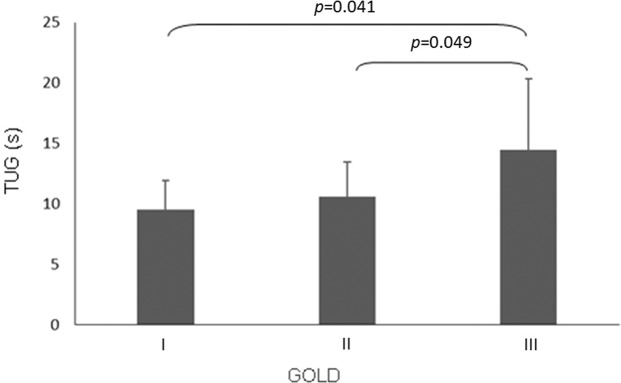
Functional performance by disease severity (one-way ANOVA, *p*=0.025; Tukey’s post hoc test (II *vs*. IV and III *vs*. IV)).

Patients were divided according to their disease staging (GOLD 2017) (01). There was no statistically significant difference between patients in terms of age (*p*=0.865), sex (*p*=0.576) or BMI (*p*=0.064). Patients classified as GOLD IV had significantly lower functional performance than GOLD II (14.46±5.88 s *vs* 9.55±2.36 s) and III (14.46±5.88 s *vs* 10.62±2.81 s), as assessed with the TUG test (Figure 2).

The frequency of sarcopenia among the 35 patients was 20% (7 patients) and was greater among individuals classified with greater disease severity, GOLD III = 4 patients (23%) and GOLD IV = 3 patients (27%), *p*=0.82.

Binary logistic regression revealed an association between a TUG time >11.2 s and sarcopenia when patients were classified by severity of disease (OR=1.55, 95% CI: 1.03-8.23, *p*=0.04).

## DISCUSSION

In this cross-sectional study, sarcopenia was associated with TUG performance after adjustment for disease severity, indicating that when disease severity is considered, COPD patients with worse TUG performance are more likely to have sarcopenia. Up to one-third of COPD patients, even in the early stages of the disease, have a loss of muscle function in their limbs (25% less strength than controls) (32).

Previous studies have shown that the frequency of sarcopenia is highly dependent on the method used for assessment and can vary from 14% to 40% in COPD patients (9,16,17). In our study, in which sarcopenia was considered to be present when HGS <30 kg for men and <20 kg for women with low muscle mass, i.e., calf circumference <30 cm and severe sarcopenia with GS ≤0.8 m/s or TUG >11.2 s, we found a frequency of 20%, showing that low muscle quantity/quality and low physical performance not only affect the elderly but also have a high percentage among patients with chronic respiratory diseases. Furthermore, when we investigated the presence of sarcopenia in our patients according to their disease staging, a greater frequency of this condition among patients with more severe COPD was found. A similar study found a higher frequency of sarcopenia in GOLD III and IV patients (15% and 20%, respectively) where patients with sarcopenia who were significantly older and had more airflow obstruction and reduced quadriceps strength, exercise capacity, functional performance, subjective and objective physical activity and health status compared with patients without sarcopenia (10).

This difference between the results of the studies may be because Costa et al. (9) used DXA, which is considered a gold standard for diagnosing sarcopenia, whereas we used calf circumference, gait speed and grip strength (33). Nevertheless, DXA often cannot be used in clinical practice because of the high cost of the equipment and the need for trained individuals to carry out the exam. The approach used in the present study has the advantage in that it requires only simple tools that can easily be used in clinical practice (e.g., a measuring tape).

Patients classified as GOLD IV had significantly lower physical performance than GOLD II and III patients assessed with the TUG test. Our GOLD IV patients completed the TUG test in 14.29 s, substantially longer than the cutoff adopted here and suggested by Mesquita et al., who showed that this test is valid for assessing functional capacity in COPD patients and that a test completion time of more than 11s indicates worse health (26). Haddad et al. proposed a cutoff >2 s to identify COPD patients at high risk for falls and reported a mean TUG time of 11.9 s in their study (34). Both values are lower than the data found in the current study.

Despite these findings, Haddad et al. did not identify a relationship between TUG performance and spirometry results or any differences between the TUG results of patients among disease stages. However, most of the patients were classified as GOLD I or II, unlike the patients from our study, who were predominantly GOLD III or IV (34).

The present study has several limitations. These include the small number of participants, possibly favoring the analysis of the stratification by the severity of the disease and the fact that muscle mass was not assessed using the gold standard. Despite the small sample, a post hoc calculation revealed a power of 0.91, i.e., a 91% chance of detecting an association between the results of the TUG and sarcopenia. Furthermore, although DXA is more accurate and reliable, it is expensive and difficult to use in clinical practice. Muscle mass was therefore assessed by measuring calf circumference, which is a simple clinical method, but not the only criteria used to screen for sarcopenia, as grip strength, gait speed and TUG were also assessed for diagnosis (35). Calf circumference may be used to measure muscle mass as a proxy for muscle quantity in settings where no other method is available (11).

## CONCLUSION

Our findings showed that a worse performance in the TUG test increases the chance of COPD patients presenting sarcopenia.

## AUTHOR CONTRIBUTIONS

Kovelis D was responsible for the literature search, data collection, study design, data analysis, manuscript preparation and review. Gomes ARS was responsible for the literature search and manuscript review. Mazzarin C was responsible for the data analysis and manuscript review. Miranda A was responsible for the data collection and study design. Valderramas S was responsible for the study design, data analysis and manuscript review.

## Figures and Tables

**Table 1:** Demographic, anthropometric, clinical and functional characteristics of the sample.

Variable	(n=35)
Age (years)	69.30±7.47
Sex F/M (n)	19/16
BMI (kg/m^2^)	24.17±5.51
Tobacco load (pack years)	42 [22-55]
FEV_1_ (% predicted)	37.93±13.64
FEV_1_/FVC	48.58±12.74
GOLD II / III / IV (n)	7/17/11
HGS (kg)	23.92±9.66
CC (cm)	34.04±4.61
GS (m/s)	0.90±0.32
TUG (s)	11.83±4.60

Data are given as the mean ± standard deviation or median [interquartile interval]; F: female; M: male; BMI: body mass index; FEV_1_: forced expiratory volume in the 1^st^ second; FVC: forced vital capacity; GOLD: Global Initiative for Chronic Obstructive Lung Disease; HGS: hand grip strength; CC: calf circumference; GS: gait speed; TUG: timed up-and-go.
